# Global, regional, and national burden and quality of care of multiple myeloma, 1990–2019

**DOI:** 10.7189/jogh.14.04033

**Published:** 2024-02-02

**Authors:** Jiawei Geng, Jianhui Zhao, Rong Fan, Zecheng Zhu, Yuchen Zhang, Yingshuang Zhu, Yichi Yang, Liying Xu, Xiangjie Lin, Kejia Hu, Igor Rudan, Peige Song, Xue Li, Xifeng Wu

**Affiliations:** 1 Department of Big Data in Health Science School of Public Health, Centre of Clinical Big Data and Analytics of The Second Affiliated Hospital, Zhejiang University School of Medicine, Hangzhou, China; 2 Centre for Global Health, School of Public Health, Zhejiang University School of Medicine, Hangzhou, China.; 3 Colorectal Surgery and Oncology, Key Laboratory of Cancer Prevention and Intervention, Ministry of Education, The Second Affiliated Hospital, Zhejiang University School of Medicine, Hangzhou, China; 4 Department of Biostatistics, Graduate School of Medicine, Hokkaido University, Sapporo, Japan; 5 Department of Social Medicine, Graduate School of Medicine, Hirosaki University, Hirosaki, Japan; 6 Department of Hematology, The First Affiliated Hospital, Zhejiang University School of Medicine, Hangzhou, China; 7 Key Laboratory of Hematologic Malignancies, Diagnosis and Treatment, Hangzhou, Zhejiang, China; 8 Centre for Global Health, Usher Institute, University of Edinburgh, Edinburgh, UK; 9 School of Public Health and Women's Hospital, Zhejiang University School of Medicine, Hangzhou, China

## Abstract

**Background:**

Multiple myeloma (MM) is the second most common haematologic malignancy, presenting a great disease burden on the general population; however, the quality of care of MM is overlooked. We therefore assessed gains and disparity in quality of care worldwide from 1990 to 2019 based on a novel summary indicator – the quality of care index (QCI) – and examined its potential for improvement.

**Methods:**

Using the Global Burden of Disease 2019 data set, we calculated the QCI of MM for 195 countries and territories. We used the principal component analysis to extract the first principal component of ratios with the combinations of mortality to incidence, prevalence to incidence, disability-adjusted life years to prevalence, and years of life lost to years lived with disability as QCI. We also conducted a series of descriptive and comparative analyses of QCI disparities with age, gender, period, geographies, and sociodemographic development, and compared the QCI among countries with similar socio-demographic index (SDI) through frontier analysis.

**Results:**

The age-standardised rates of MM were 1.92 (95% uncertainty interval (UI) = 1.68, 2.12) in incidence and 1.42 (95% UI = 1.24, 1.52) in deaths per 100 000 population in 2019, and were predicted to increase in the future. The global age-standardised QCI increased from 51.31 in 1990 to 64.28 in 2019. In 2019, New Zealand had the highest QCI at 99.29 and the Central African Republic had the lowest QCI at 10.74. The gender disparity of QCI was reduced over the years, with the largest being observed in the sub-Saharan region. Regarding age, QCI maintained a decreasing trend in patients aged >60 in SDI quintiles. Generally, QCI improved with the SDI increase. Results of frontier analysis suggested that there is a potential to improve the quality of care across all levels of development spectrum.

**Conclusions:**

Quality of care of MM improved during the past three decades, yet disparities in MM care remain across different countries, age groups, and genders. It is crucial to establish local objectives aimed at enhancing MM care and closing the gap in health care inequality.

Multiple myeloma (MM), which manifests as unregulated clonal plasma cell proliferation in the bone marrow, is the second most common clonal plasma cell cancer (plasma cancer evolved through reiterative clonal expansion), accounting for around 10% of all haematologic malignancies [1–3]. Up to 63% of diagnosed patients are >65 years old [4]. The alterations in the immune system linked to ageing, known as immunosenescence, have been associated with tumour immunosurveillance and the subsequent development of MM in older individuals [5]. Patients with MM are reported to have the highest burden of physical symptoms among all haematologic malignancies, including destructive bone lesions and pain, anaemia, hypercalcemia, repeated infections, and kidney injury [6,7]. The advanced treatment paradigms, including autologous hematopoietic cell transplantation, immunomodulatory drugs, targeted monoclonal antibodies, and proteasome inhibitors, have significantly increased the five-year survival rate of MM, which is now approaching 50% [8–10]. Despite efforts to improve the management across the care continuum of MM, the increasing trends of MM incidence and prevalence continuously challenge the care ability and raise substantial concerns about the quality of care for MM worldwide [11,12].

Quality of care is defined as the extent to which health care services increase the desired health outcomes for the population [13]. It has received increasing attention in the context of Sustainable Development Goals (SDGs), which have called for improvements in care performance on safety, effectiveness, patient-centeredness, timeliness, efficiency, and equity [14]. However, previous studies have primarily been limited to individual-level health outcomes [15–17] and health system assessment or structure improvement in developed countries [18–20], with few reporting on the quality of care of MM. Although such studies have provided insights into the effects of particular care models in specific contexts, they have had limited policy relevance and generalisability across developing countries. Moreover, health care inequity has been a major problem in health care systems for a long time. Survival data directly reflect cancer prognosis and progress in cancer control [21]. Mitigating inequality in survival generally leads to improvements in average cancer care [22]. However, due to the lack of direct survival information on MM for most countries, few studies systematically quantified and compared the possible disparity of health care at the global level [19].

The quality of care index (QCI) was conceptualised in 2021 as an indicator for assessing the quality of care of disease [23]. It has been validated among various cancers, including MM (*r* = 0.85), using another health system indicator called the health care access and quality index (HAQ), which is an index of amenable mortality designed to reflect effective access to health care [24,25]. To understand the gains, progress, and potential improvements in quality of care of MM, we examined the QCI at the global, regional, and national levels from 1990 to 2019. In recognising the overall health care performance for MM over time and identifying gaps that can be addressed with available resources, targeted and actional health strategies can be more effectively developed, especially in countries and territories experiencing lagging performance.

## METHODS

### Data source

We retrieved data from the Global Burden of Disease Study 2019 (GBD 2019), which examined incidence, mortality, prevalence, years of life lost (YLLs), years of life living with disability (YLDs), and disability-adjusted life years (DALYs) related to various diseases, stratified by age, sex, and location. Detailed methods for the GBD study and cancer estimates have been reported elsewhere [26]. MM was determined per the International Classification of Disease, 9th (ICD-9) or 10th edition (ICD-10) codes (nonfatal cause: ICD-10 – C88-90.32, ICD-9 – 203-203.9; death cause: ICD-10 – C88-90.9, ICD-9 – 203-203.9) [27]. We complied and systematically analysed relevant data from a total of 21 grouped global burden regions, and seven super regions containing 195 countries and territories (excluding Cook Islands, Monaco, Nauru, Niue, Palau, Saint Kitts and Nevis, San Marino, Tokelau, and Tuvalu due to insufficient data).

Countries were further classified based on the socio-demographic index (SDI), a composite measure calculated based on the total fertility rate in a population younger than 25, educational attainment for those aged 15 and older, and lag-distributed income per capita [28]. The SDI facilitates a comparison of social and economic development status by geography and across different periods. Despite countries and territories varying in their stages of the MM epidemiology, diagnosis, and treatment across different resource contexts, the SDI classification enhances the policy relevance of the results [26,27,29]. SDI was divided into quintiles and termed as high, high-middle, middle, low-middle, and low with the cut points of 0.455, 0.608, 0.690, and 0.805, respectively [27].

### Statistical analyses

#### Measures of burden

We used descriptive statistics to gain an overview of the MM in incidence, mortality, and DALYs, generating their 95% uncertainty intervals (UI) by 1000 posterior draws. We calculated estimated annual percentage changes (EAPC) and 95% confidence intervals (CI) of age-standardised incidence and mortality rates by using a log-linear regression model ((*e^β^* − 1) × 100) to reflect secular trends of disease burden in the past three decades. To identify geographies with identical shifts in the disease burden of MM over the years, we further grouped 195 countries and territories according to the EAPCs of the age-standardised prevalence rate and their corresponding 95% confidence intervals (CIs) by conducting the hierarchy cluster analysis (HCA) using Euler distance and complete linkage. Additionally, we applied the Bayesian age-period-cohort (BAPC) model with integrated nested Laplace approximations [30] to predict the number of cases by computing corresponding age-sex-specific rates of incidence and mortality from 2020 to 2030. Data regarding the world population projections and the world standard population were based on Institute of Health Metrics population forecasts and the GBD 2019, respectively [31].

#### Estimating QCI

We computed four indicators to generate QCI for MM: the ratio of mortality to incidence (MIR), prevalence to incidence, DALYs to prevalence, and YLLs to YLDs. These four ratios were combined to compare the health care outcomes in the same situation. We then extracted the first principal component (which we regarded as the QCI) from these four indicators by using the principal components analysis (PCA). We calculated the QCI score and rescaled it into a range of 0 to 100, with higher scores indicating higher quality of care. Additional details regarding QCI, including information on four indicators’ rationale, performance of the first principal components, and the related validation study can be found in the **Online Supplementary Document**. We also generated the value of gender disparity ratio (GDR), i.e. the QCI score for females divided by the QCI score for males, to compare the gender disparity in the care of MM. Both QCI and GDR were detailed in previous studies [23,25,32–35].

We described the specific QCI and its temporal trend by sex and age and presented the corresponding GDR as appropriate. We also calculated the relative changes in age-standardised QCI for each country between 1990 and 2019 by the formula ((age-standardised QCI in 2019 − age standardised QCI in 1990)/age-standardized QCI in 1990)). Additionally, we performed Pearson correlation to examine the associations of QCI and GDR with SDI across the development spectrum on the national, regional, and global levels.

#### Frontier analysis

To determine the highest potential of age-standardised QCI at given values of SDI for all countries and territories, we conducted the frontier analysis using data envelope analysis (DEA) under the free disposal hull (FDH) model. DEA is a performance measurement technique employed to assess the relative efficiency of the decision-making units (DMUs), which in this context comprised all countries and territories. We used the method to evaluate the efficiency of generated output (age-standardised QCI) from a given set of input (SDI) to identify the highest attainable QCI based on the sociodemographic development of countries and territories.

Following previous studies, we applied FDH model to determine the frontier as it can relax the convexity consumption [36]. We bootstrapped samples of data with replacement from 1990 to 2019 for 1000 times to account for the uncertainty of the frontier line. During this process, we used super efficiency DEA to address the influence of outliers [37]. Super efficiency DEA leaves out each point to calculate the efficiency score. If the obtained efficiency score for the removal point exceeded 1, we deemed the removed point as the super-efficient point (i.e. outlier). We repeated the leave-one-out method for all points in the bootstrapped sample to detect super-efficiency point which we removed before generating the frontier [24,38]. We computed the mean frontier of each SDI and then used Loess regression with a local polynomial degree of 1 and span of 0.2 to generate the smoothed frontier. To show the unrealised potential of QCI in 2019, we also computed the absolute difference between the frontier and the observed QCI, which is termed as the effective difference [24,38–40]. The effective difference indicated the extent to which a country or a territory deviates from the optimal state. We defined a *P*-value <0.05 as statistically significant. All statistical analyses were conducted in R, version 4.1.2 (R Core Team, Vienna, Austria).

## RESULTS

### Overview

The global age-standardised incidence, mortality, and DALYs of MM in 2019 were 1.92 (95% UI = 1.68, 2.12), 1.42 (95% UI = 1.24, 1.52), 30.26 (95% UI = 26.58, 32.9) per 100 000 persons, respectively (**Table 1**). The age-standardised incidence increased for both sexes with an EAPC of 0.25% (95% CI = 0.15, 0.35), while the mortality and DALYs decreased with EAPCs of −0.07% (95% CI = −0.15, 0.01) and −0.16% (95% CI = −0.23, −0.09), respectively. MM was more prevalent in males than females, and EAPCs of all indicators (incidence, deaths, and DALYs) were more pronounced in males as well.

**Table 1:** All age cases and age-standardised incidence rates, deaths, and DALYs for multiple myeloma in 1990 and 2019

	All-ages cases, n (95% UI)			Age-standardised rates per 100 000 (95% UI)		
**Measure**	**Male**	**Female**	**Total**	**Male**	**Female**	**Total**
1990						
*Incidence*	33 435.09 (29 581.9, 38 797.3)	32 505.45 (29 374.32, 38 019.52)	65 940.53 (60 779.82, 74 058.8)	1.97 (1.74, 2.25)	1.55 (1.4, 1.81)	1.73 (1.59, 1.93)
*Deaths*	25 880.15 (22 674.21, 30 034.77)	25 982.14 (23 553.98, 30891)	51 862.29 (47 709.89, 58 979.91)	1.59 (1.41, 1.85)	1.26 (1.13, 1.49)	1.4 (1.28, 1.58)
*DALYs*	638 159.74 (555 968.87, 752 900.42)	585 202.28 (530 614.24, 691 914.55)	1 223 362.02 (1 122 711.78, 1 412 932.34)	34.34 (30.06, 40.1)	27.34 (24.75, 32.23)	30.52 (27.98, 35)
2019						
*Incidence*	84 516.24 (70 924.46, 94 909.94)	71 171.42 (60 343.34, 80 140.24)	155 687.66 (136 585.38, 172 576.69)	2.28 (1.91, 2.56)	1.62 (1.38, 1.83)	1.92 (1.68, 2.12)
*Deaths*	60 445.16 (50 723.29, 67 055.55)	53 029.2 (45 148.58, 58 252.4)	113 474.36 (99 527.45, 121 735.03)	1.68 (1.4, 1.84)	1.21 (1.03, 1.33)	1.42 (1.24, 1.52)
*DALYs*	137 6624.09 (115 0624.7, 1567 825.93)	112 0581.22 (967 699.7, 1 243 740.92)	2 497 205.31 (2 190 467.04, 2 722 668.53)	35.51 (29.77, 40.03)	25.67 (22.15, 28.48)	30.26 (26.58, 32.9)
**1990–2019**	**Percentage of relative change in cases**			**Estimated annual percentage change (95% CI)**		
Incidence	152.78	118.95	136.10	0.43 (0.33, 0.53)	0.02 (−0.09, 0.13)	0.25 (0.15, 0.35)
Deaths	133.56	104.10	118.80	0.10 (0.03, 0.17)	−0.28 (−0.38, −0.19)	−0.07 (−0.15, 0.01)
DALYs	115.72	91.49	104.13	0.01 (−0.05, 0.08)	−0.38 (−0.46, −0.30)	−0.16 (−0.23, −0.09)

DALYs – disability-adjusted life years, EAPC – estimated annual percentage change

While we observed significant downward trends for age-standardised mortality (EAPC = −0.28; 95% CI = −0.38, −0.19) and DALYs (EAPC = −0.38; 95% CI = −0.46, −0.30) for females, the trends differed for males, with increasing mortality (EAPC = 0.10; 95% CI = 0.03, 0.17) and stable trends for DALYs (EAPC = 0.01; 95% CI = −0.05, 0.08). The hierarchy cluster analysis (HCA) of EAPCs of age-standardised prevalence rate identified three categories among 195 countries and territories – those that ‘remained stable or had a low increase’ (n = 104), those with a ‘middle increase’ (n = 78), and those with a ‘high increase’ (n = 13). Meanwhile, the disease burden of MM will continue to increase. It is estimated that the number of incident cases and deaths of MM will continue to increase and reach 215 313.37 (95% CI = 173 614.57, 257 012.86) and 158 095.13 (95% CI = 128 473.49, 187 717.43) in 2030, representing a 1.38 and 1.39-fold increase, respectively (Table S1 and Figures S1–2 in the **Online Supplementary Document**).

### Global levels and trends of quality of care index and gender disparity ratio

**Figure 1:**
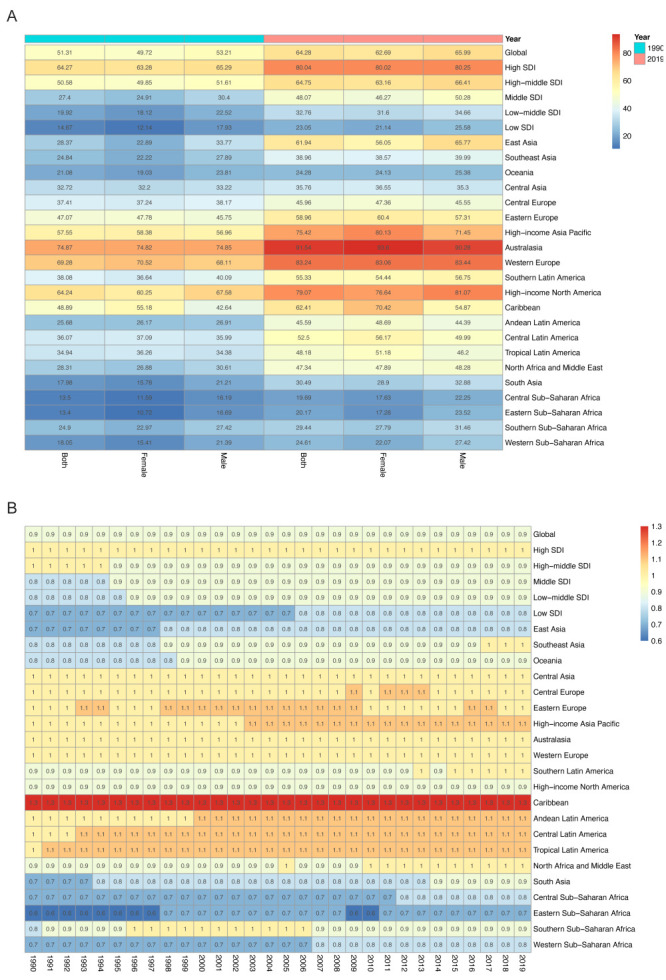
Heatmap of age-standardised QCI and GDR. **Panel A.** QCI in 1990 and 2019 in global and regions. The color scale represents the QCI from 0 depicted in blue to 100 depicted in red. Annotation in green represents the year 1990, and red represents the year 2019. The numbers shown in each block are QCI. **Panel B.** GDR from 1990 to 2019 in global and regions. The colour scale represents the GDR from 0.6 depicted in blue to 1.3 depicted in red. SDI – socio-demographic index.

**Figure 2:**
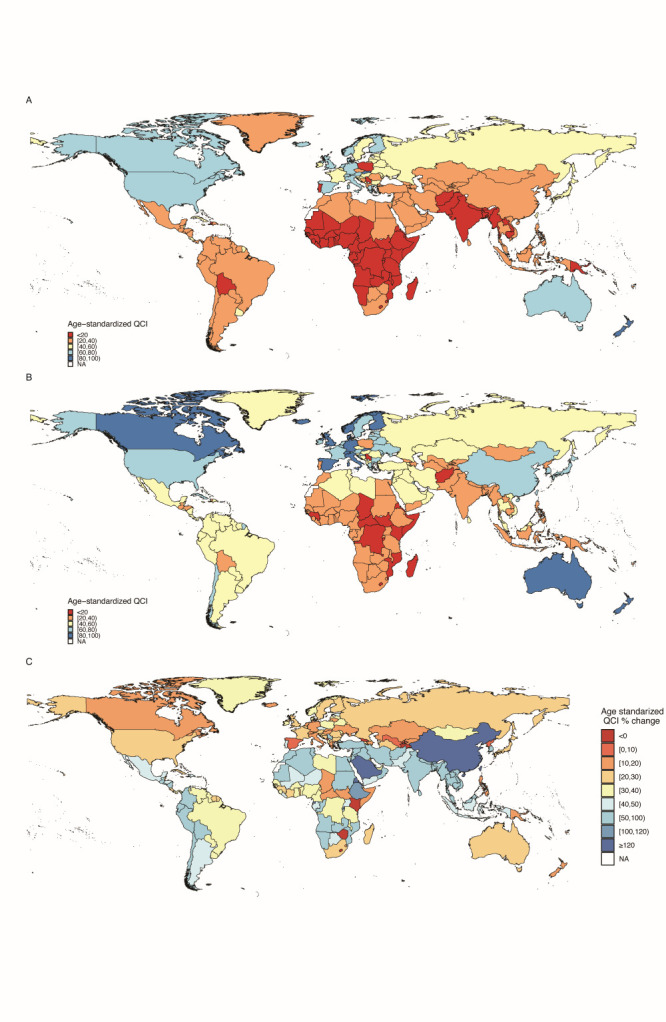
The map of the age-standardised QCI at the national level. **Panel A.** QCI in 1990. **Panel B.** QCI in 2019. **Panel C.** Relative percentage change in age-standardised QCI for both sexes between 1990 and 2019. QCI – quality of care index.

In 2019, the global age-standardised QCI of MM was 62.69 in females, 65.99 in males, and 64.28 overall (i.e. both sexes). At the regional level, Australasia showed the highest QCI (91.54), while Central sub-Saharan Africa had the lowest (19.69) (**Table 2** and **Figure 1**, Panel A). Among 195 countries and territories, New Zealand achieved the highest score of QCI (99.29), while the Central African Republic had the lowest score (10.74). Compared with 1990, the gap between the lowest QCI and highest QCI values at the regional level increased from 78.96 to 88.55 in 2019 (**Figure 2**, Panels A and B).

**Table 2:** Age-standardised QCI and GDR in 1990 and 2019

	1990				2019			
	**QCI**			**GDR**	**QCI**			**GDR**
	**Both**	**Female**	**Male**		**Both**	**Female**	**Male**	
**Global**	51.31	49.72	53.21	0.93	64.28	62.69	65.99	0.95
**21 global health regions**								
East Asia	28.37	22.89	33.77	0.68	61.94	56.05	65.77	0.85
Southeast Asia	24.84	22.22	27.89	0.80	38.96	38.57	39.99	0.96
Oceania	21.08	19.03	23.81	0.80	24.28	24.13	25.38	0.95
Central Asia	32.72	32.2	33.22	0.97	35.76	36.55	35.3	1.04
Central Europe	37.41	37.24	38.17	0.98	45.96	47.36	45.55	1.04
Eastern Europe	47.07	47.78	45.75	1.04	58.96	60.4	57.31	1.05
High-income Asia Pacific	57.55	58.38	56.96	1.02	75.42	80.13	71.45	1.12
Australasia	74.87	74.82	74.85	1.00	91.54	93.6	90.28	1.04
Western Europe	69.28	70.52	68.11	1.04	83.24	83.06	83.44	1.00
Southern Latin America	38.08	36.64	40.09	0.91	55.33	54.44	56.75	0.96
High-income North America	64.24	60.25	67.58	0.89	79.07	76.64	81.07	0.95
Caribbean	48.89	55.18	42.64	1.29	62.41	70.42	54.87	1.28
Andean Latin America	25.68	26.17	26.91	0.97	45.59	48.69	44.39	1.10
Central Latin America	36.07	37.09	35.99	1.03	52.5	56.17	49.99	1.12
Tropical Latin America	34.94	36.26	34.38	1.05	48.18	51.18	46.2	1.11
North Africa and Middle East	28.31	26.88	30.61	0.88	47.34	47.89	48.28	0.99
South Asia	17.98	15.78	21.21	0.74	30.49	28.9	32.88	0.88
Central Sub-Saharan Africa	13.50	11.59	16.19	0.72	19.69	17.63	22.25	0.79
Eastern Sub-Saharan Africa	13.40	10.72	16.69	0.64	20.17	17.28	23.52	0.73
Southern Sub-Saharan Africa	24.9	22.97	27.42	0.84	29.44	27.79	31.46	0.88
Western Sub-Saharan Africa	18.05	15.41	21.39	0.72	24.61	22.07	27.42	0.8
**SDI quintile**								
High SDI	64.27	63.28	65.29	0.97	80.04	80.02	80.25	1.00
High-middle SDI	50.58	49.85	51.61	0.97	64.75	63.16	66.41	0.95
Middle SDI	27.4	24.91	30.4	0.82	48.07	46.27	50.28	0.92
Low-middle SDI	19.92	18.12	22.52	0.80	32.76	31.60	34.66	0.91
Low SDI	14.67	12.14	17.93	0.68	23.05	21.14	25.58	0.83

QCI – quality of care index, GDR – gender disparity ratio, SDI – sociodemographic index

**Figure 3:**
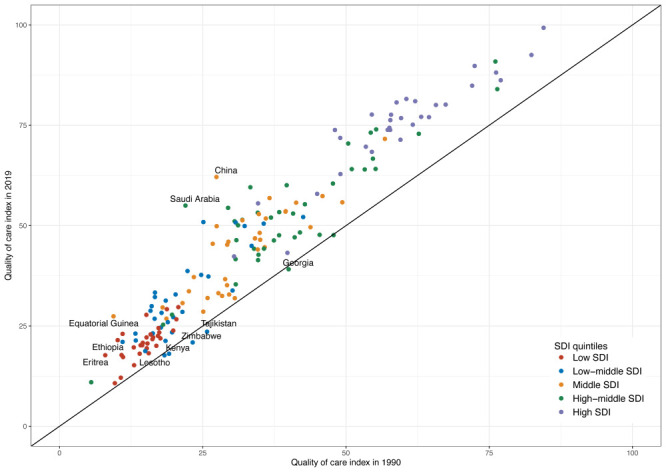
The comparison of the QCI between 1990 and 2019 among 195 countries and territories. All countries and territories are color-coded by the SDI quintile of countries in 2019. The top five countries (Equatorial Guinea, Saudi Arabia, China, Eritrea, and Ethiopia) with the largest and the last five countries (Zimbabwe, Tajikistan, Kenya, Lesotho, and Georgia) with the smallest relative percentage change from 1990 to 2019 are labelled. SDI – socio-demographic index.

From 1990 to 2019, the overall age-standardised QCI of MM had an increasing trend in most countries globally, and the improvement was more pronounced in areas of East Asia, South Asia, Africa, and Latin America (**Figure 2**, Panel C). Among the 21 GBD regions, the fastest growth occurred in East Asia, where the QCI increased from 28.37 to 61.94, with the highest relative change of 118.33% over the past 30 years. However, Central Africa had the smallest improvement in MM QCI (from 32.72 to 35.76, an increase of 9.29%). At the national level, Equatorial Guinea had the highest relative change (190.87%, from 9.42 to 27.4,), followed by Saudi Arabia (by 150.23%, from 21.98 to 55) and China (by 126.80%, from 27.39 to 62.12). However, six countries had decreasing trends in QCI by 2019: Georgia (−2.15%), Kenya (−5.44%), Lesotho (−3.49%), Montenegro (−0.44%), Tajikistan (−8.47%), and Zimbabwe (−10.07%) (**Figure 3**).

**Figure 4:**
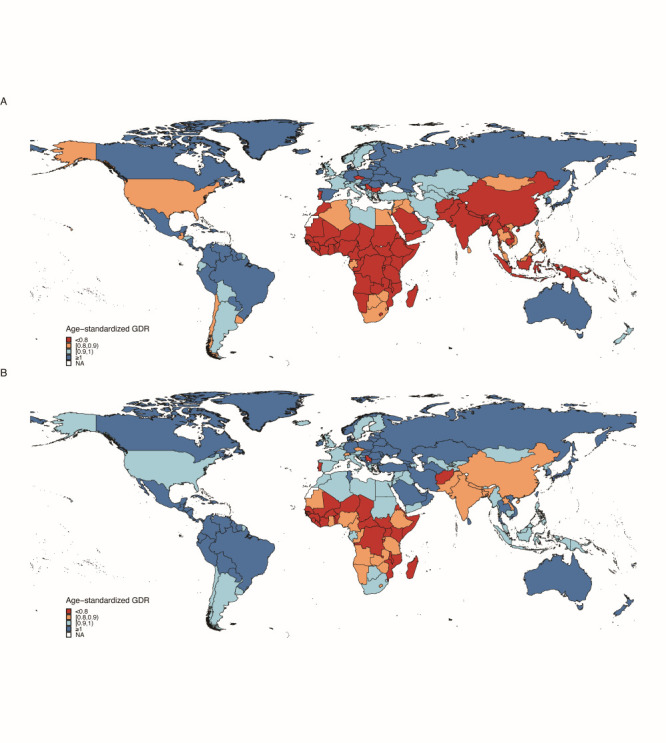
The map of age-standardised GDR at the national level. **Panel A.** GDR in 1990. **Panel B.** GDR in 2019. GDR equal to 1 denotes absolute sex equity, lower than 1 denotes better quality of care for males, and more than 1 denotes better quality of care for females. GDR – gender disparity ratio.

Globally, from 1990 to 2019, the GDR maintained an increasing trend and reached 0.95 in 2019. Caribbean and Eastern Sub-Saharan Africa were regions with the highest and lowest GDRs over years, and had a GDR of 1.28 and 0.73 in 2019, respectively (Figure 1B). Nationally, age-standardized GDR ranged from 0.39 (Serbia) to 1.38 (Suriname) in 2019, while the value ranged from 0.05 (Serbia) to 1.40 (Poland) in 1990 (**Figure 4**). Gender disparity which is in favor of better care in males than females in QCI of MM was mitigated globally during the past three decades, especially in Asia. Nevertheless, the gender discrepancy of MM in QCI remained significant among geographic areas located in Sub-Saharan Africa (**Figure 4**, Panels A and B).

### The quality of care index and gender disparity ratios by socio-demographic index

**Figure 5:**
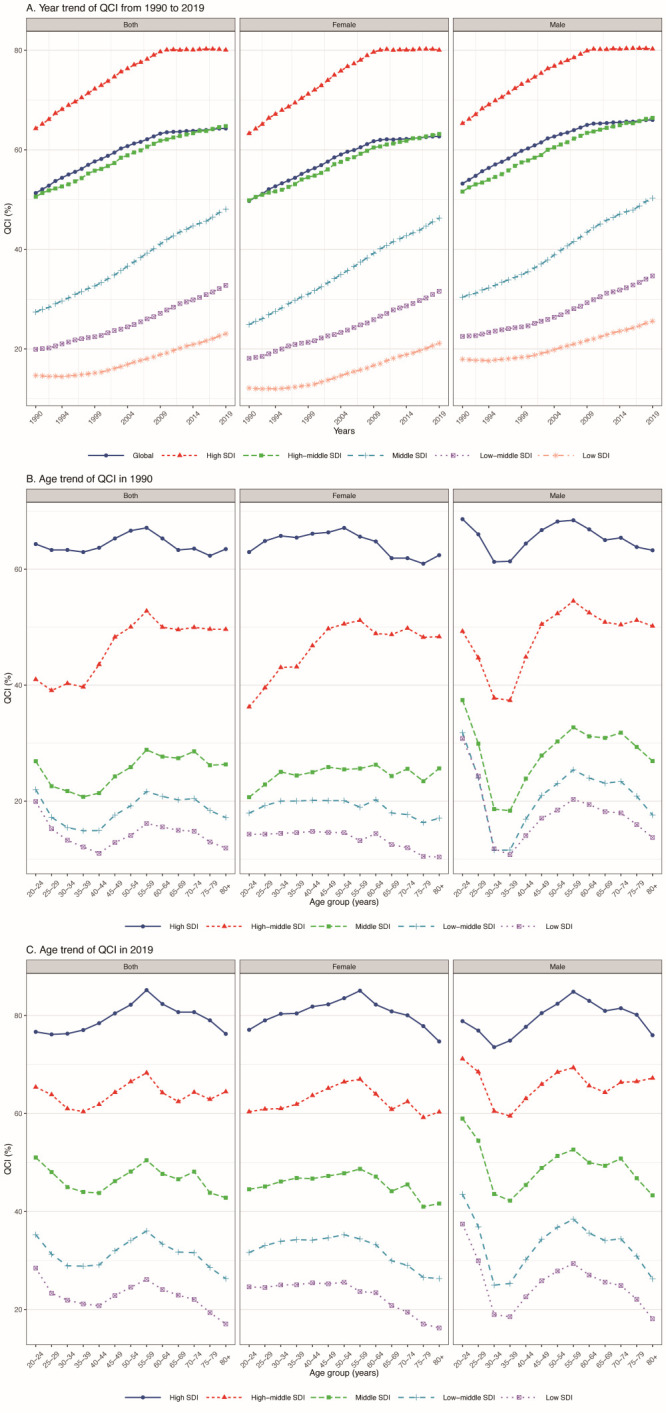
QCI by SDI quintiles. **Panel A.** Temporal QCI from 1990 to 2019. **Panel B.** Age trend of QCI in 1990. **Panel C.** Age trend of QCI in 2019. QCI – quality of care index, SDI – socio-demographic index.

QCI increased to 80.04 in areas with high SDI in 2019, while it was 23.05 for those regions with the lowest SDI. Age-standardised QCI showed comparable patterns in different SDI quintiles, where we observed increasing trend in all SDI quintiles over the years. The most gains in QCI took place in middle SDI regions, followed by high SDI quintile over the past three decades (**Figure 5**, Panel A). QCI was positively correlated with the higher SDI (*r* = 0.829, *P* < 0.001) (Figures S3A and S3B in the **Online Supplementary Document**).

To gain an overview on the age-specific QCI across SDI regions, we delineated the evolving trends of QCI values. For high and high-middle SDI areas, the MM QCI increased overall in the population under the 55-59 age group in 2019, but had dropped since then. For middle to lower SDI quintiles, the QCI was relatively stable and maintained in a relatively low status (**Figure 5**, Panel B). In 2019, all SDI quintiles showed a steady increase before peaking within the 55–59-year-old group in 2019, and still underwent a declined for patients in older groups for both sexes (**Figure 5**, Panel C). However, a notable gap of QCI between males and females still exist. While the QCI was almost comparable between the sexes in the high SDI regions, it was higher overall in males than females across all age groups in other ones. Of particular concern are the gaps in middle- to lower-level quintiles. Unlike females, males experienced a distinct pattern within the 30–39-year-old age group in both 1990 and 2019; here we observed a drop in QCI which resulted in the lowest QCI values for males across all SDI regions.

We observed a positive correlation between GDR and SDI among countries and subnations (*r* = 0.554, *P* < 0.001). The low SDI quintile showed the most pronounced increase in GDR over time (GDR = 0.83 in 2019), which shortened the gap with others and reached to 0.83 in 2019. Young age groups (30 to 50 years) had a higher QCI value for females than male, but reversals occurred for those 50 years or more in all except for the high SDI quintile, where the GDR levelled off near 1 (Figure S4, Panels A and B; Figure S5, Panels A-C in the **Online Supplementary Document**).

**Figure 6:**
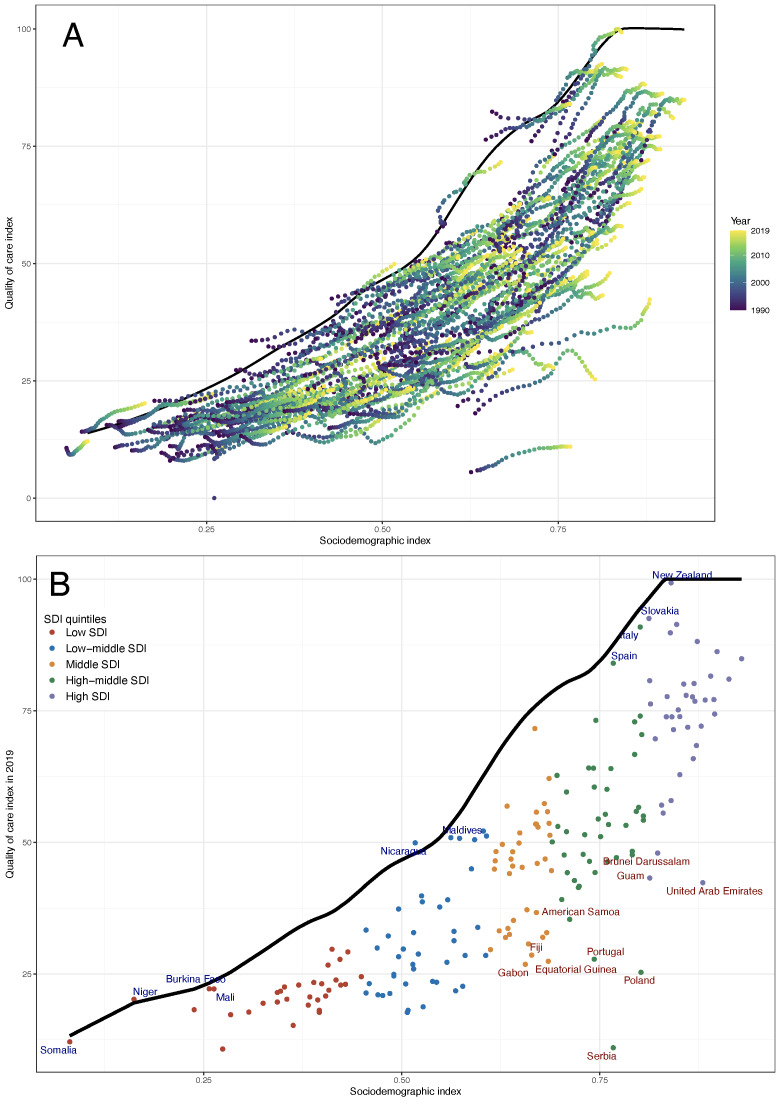
Frontier analysis based on SDI and age-standardized QCI. **Panel A.** QCI from 1990 to 2019. The colour scale represents the years from 1990 depicted in purple to 2019 depicted in yellow. **Panel B.** QCI in 2019. Each circle denotes the QCI and levels of SDI for a given geography year. The frontier is depicted in a solid black colour. All countries and territories are color-coded by the SDI quintile of countries in 2019. The top 10 countries with the least effective difference (the largest gap between the observed QCI and the frontier QCI) are labelled in red (Nicaragua, Niger, New Zealand, Burkina Faso, Somalia, Mali, Maldives, Chad, Italy, and Slovakia), and the top 10 largest effective difference are labelled in blue (Montenegro, Fiji, Gabon, Brunei Darussalam, Equatorial Guinea, Guam, Portugal, United Arab Emirates, Poland, and Serbia). SDI – socio-demographic index.

In the frontier analysis, the optimal achievable QCI rose with the SDI increase (**Figure 6**, Panels A and B and in Table S2 in the **Online Supplementary Document**). The effective difference in a given SDI in 2019 showed that the top 10 countries with the highest unrealised potential were Montenegro, Fiji, Gabon, Brunei Darussalam, Equatorial Guinea, Guam, Portugal, United Arab Emirates, Poland, and Serbia, with effective differences ranging from 45.53 to 77.3. The top 10 countries that achieved better QCI compared with their comparable SDI were Nicaragua, Niger, New Zealand, Burkina Faso, Somalia, Mali, Maldives, Chad, Italy, and Slovakia, with an effective difference <4.15.

## DISCUSSION

We systematically assessed the global disease burden and quality of care for MM by employing a novel parameter (QCI). Our analysis included the latest large-scale GBD 2019 data from 21 global health regions and five SDI quintiles containing 195 countries and territories which provided evidence for us to direct efforts towards improvement in specific areas. Our findings highlighted the care disparity by sex, age, and geographic regions over time. Combined with the frontier analysis, this study offers guidance for future resource allocation across the care continuum of MM, since the gap between observed QCI and unrealised frontier could be potentially lessened by using countries’ available sociodemographic resources.

We found an increase in the QCI value of MM for nearly all countries between 1990 and 2019, with a global increase of 25.28%, indicating that the quality of care of MM survivors had improved greatly. This can be attributed to the great progress in understating the disease mechanisms, improved diagnosis, and advanced treatments, and the general global health care enhancement over the past three decades. As the pre-malignant stage of MM is asymptomatic and the disease manifestations are nonspecific, delays in MM diagnosis are common [41,42]. One significant improvement in the diagnosis of MM is the updated diagnostic criteria revised by The International Myeloma Working Group (IMWG) in 2014, which improved the diagnostic precision and earlier intervention on patients with MM through identification on the end-organ damages (hypercalcemia, renal failure, anaemia, and bone lesions) and validated biomarkers for high-risk group detection [43]. Therapeutic strategies, including alkylating agents, corticosteroids, immunomodulatory drugs, and proteasome inhibitors, monoclonal antibody for MM also evolved rapidly, which substantially increased the number of patients who attained deep remission and favourable prognoses [44]. Likewise, a notable rise in the median survival period from 3–5 to 8–10 years had been achieved in the last decade [45].

We observed a positive correlation between SDI and QCI, with improvements observed across all SDI quintiles over the years. While middle SDI countries experienced the swiftest rise in QCI, the rapid increase in QCI in high SDI areas further exacerbated pre-existing gaps. These findings suggest that care resources remain disproportionally concentrated in higher SDI regions. One of the predominant reasons for this phenomenon might related to inadequate diagnosis [11]. For example, MM is more likely to be undetected or misdiagnosed in the early stage, resulting in further deterioration in the survival outcome for patients due to lack of timely diagnoses [29]. The shortage of pathologists in lower SDI region is also a contributing factor; the pathologist-to-population ratio in sub-Saharan Africa was 1:100 000 000, which is unacceptably low compared with 1:25 000 and 1:36 000 in the USA and UK [46]. Besides, the availability of treatments and standard of care usually lag in lower resource settings, further contributing to the disparity. However, the disease burden of MM in middle to low SDI regions was outpaced the high SDI areas due to ageing, improving diagnostic capability, and growing concern on metabolic risk factors [11,47]. Per our estimates, the global burden of MM will continue to rise, highlighting the significant challenge on MM quality of care. Results have indicated a positive correlation between SDI and QCI, and improvements took place in every SDI quintile over year [11,29]. As we mentioned previously, there are opportunities to improve the QCI of MM patients across the development spectrum based on the acquired socioeconomic resources. Countries in low SDI regions which performed well and appropriately used their resources might serve as examples for those also in comparable constraint resource settings. Further studies could identify the contributors to their successes and accessible approaches to reduce the unnecessary suffering of MM.

Notably, low- and middle-income countries (LMICs) located in Latin America, South Asia, and most areas in Africa experienced more gains in QCI relative to 1990; however, the gap in QCI between the highest and lowest countries widened from then to 2019, possibly indicating an unbalanced development of care resources worldwide. Due to the socioeconomic disparity, certain areas may experience insufficient diagnostic and treatment capacity or unaffordability of treatment for patients, impeding the delivery of optimal quality of care [11]. To compensate for the disproportional resources in health care infrastructure and treatment, resources-stratified guidelines for diagnosis and treatment of MM were put into practice many years ago [48]. These efforts address the detection and treatment of in LMICs to some extent, increasing access to and making essential care more affordable for patients [29]. However, the challenges of the widening gaps remained. In fact, inequality has been exacerbated with the emergence of new, expensive therapy [49], resulting in concerns on the limited availability and applicability of treatment. A recent review synthesised 18 pivotal MM clinical trials from 2005 to 2019 and reported a lag for treatment approval in LMICs, which were likewise under-represented in clinical trials [50]. Europea and Central Asia had the highest trial approval rates for MM (with a time lag of 8.3 months), while Latin America and sub-Saharan Africa did not even have a regimen approval for the trials in which they participated [50]. For example, lenalidomide and bortezomib, listed as the standard care of MM are still not approved in some LMICs located in Africa and Mid-eastern Asia [29]. Developing and innovating new combinations of treatment regimens, such as the incorporation of low-cost agents, could be a cost-effective approach to benefit populations in unprivileged situations [51].

Apart from the unprivileged regions, we found an inequity of care resources in vulnerable populations, such as older patients and females. Based on our findings, QCI dropped in patients aged over 60 years old across all SDI regions. To date, stem cell transplantation and new drug therapy are offered preferentially to relatively young patients, as they are more likely to have a good prognosis for their better physical health status compared with older patients. Meanwhile, existing treatment was reported to be invalid or suboptimal for the older population [52,53]. Therefore, to potentially solve the age disparity in MM care, new regimens with lower toxicity and frailty-adapted therapy are required [54,55]. We also noticed a drop in the quality of care for MM among the younger aged group (30–39 years) for males. The results align with a previous study where significantly young patients were mostly male, although the reasons remain to be elucidated [56]. Additionally, our results showed the marked gender disparity in MM care, particularly in sub-Saharan Africa. Sex disparities in MM care and health outcomes require further investigation [57]. Since males are more likely to developed MM than females, the results could emphasise worse care access and prognostic outcomes for the latter group [55]. This may be partly traced to the socio-cultural inequality, lack of awareness of the disease, and improper health-seeking behaviour among women, suggesting a need for gender empowerment [58]. Also, sex-stratified medicine to improve the treatment and care access for females might also be a promising approach [59].

Our shared the same limitations as other GBD-based studies. Although the latest GBD study improved the precision of indicator imputation and estimation, it is still limited by the quality of available data and is time-lagged in data accessibility [27]. Also, the data acquired might biased by undiagnosed MM cases. We also could not account for the uneven development within a country or at a subnational level, since we relied on data at a macro-level data [38]. Furthermore, we attempted to provide information on the status, gains, and opportunities on the quality of care targeting on the disease of MM by using QC as a novel indicator. However, due to the limited availability of confounding factors for stratification and adjustment in GBD2019, we cannot exclude the potential impact of unreported confounders on QCI. We did not address other possible confounders that influence the QCI results, such as the incidence variation in different ethnicity and heterogeneity of MM subtypes in different geographics [6].

## CONCLUSIONS

This study provides evidence on the quality of care of MM at the global level over time, suggesting an overall improvement but a notable remaining gap in care equity over the past 30 years. Variations in demographic and geographic were significant; the high quality of care leaned towards countries and regions with high socioeconomic development. Females and older patients with MM were still vulnerable population in view of access favourable care. We also observed unrealised potential to increase the quality of care across the SDI spectrum. Informed by our findings on the progress and inequity gaps on MM care, health policymaking could focus on improving the diagnosis and treatment accessibility and applicability based on countries’ own socioeconomic resources, especially in those with lagged performance. To mitigate disparities in global MM care, further studies are needed to determine the driving factors of high-quality care and obstacles leading to lagging performance.

## Additional material


Online Supplementary Document
