# Supplementary material for: Global, regional, and national burden and quality of care of multiple myeloma, 1990–2019
Source: J Glob Health. 2024 Feb 2;14:04033. doi: 10.7189/jogh.14.04033 (PMC10832550; doi:10.7189/jogh.14.04033)
Supplement: Online Supplementary Document [file jogh-14-04033-s001.pdf]

Supplementary Method

Supplementary Tables and Figures

Table S1. Estimated number of multiple myeloma incidence and deaths from 2020 to 2030

Table S2. Frontier age standardized quality of care index (QCI), and effective difference by countries and territories.

Figure S1. The hierarchical clustering analysis of estimated annual percentage changes (EAPC) of age-standardized prevalence rate for multiple myeloma among 195 countries and territories.

The blue cluster represents remained stable or low increase (104 geographies); the red cluster represents middle increase (78 geographies); the green cluster represents high increase (13 geographies).

Figure S2. Trends in multiple myeloma sex-specific incidence and deaths of global from 1990-2030.

Dots represent observed number of incident cases and deaths; Square crosses represent predicted number of incident cases and deaths by Bayesian age-period-cohort models (BAPC); For reference, shading represents 95% confidence interval of the predicted results.

Figure S3. The association between quality of care index (QCI) and socio-demographic index (SDI). **Panel A**. Countries. **Panel B**. Regions.

The smoothed line in black depicted by using loess regression shows the possible relationship between SDI and QCI; The Pearson correlation coefficient between SDI and QCI on national level was 0.829.

Figure S4. The association between gender disparity ratio (GDR) and socio-demographic index (SDI). **Panel A**. Countries. **Panel B**. Regions.

The smoothed line in black depicted by using loess regression shows the possible relationship between SDI and GDR; The Pearson correlation coefficient between SDI and GDR on national level was 0.554.

Figure S5. Gender disparity ratio (GDR) by socio-demographic index (SDI) quintiles. **Panel A.** Temporal GDR from 1990 to 2019. **Panel B.** Age trend of GDR in 1990. **Panel C.** Age trend of GDR in 2019.

Supplementary Method

1. Assessment of quality of care index (QCI) [1-4]

Markers of QCI

Mortality-to-incidence ratio (MIR): It was calculated by dividing mortality by incidence, which is sometimes act as a proxy for 5-year survival due to cancers. The higher the MIR value is, the worse the care is.

DALYs-to-Prevalence ratio: It was calculated by dividing disability adjusted life years (DALYs) by prevalence. DALYs are the sum of years of life lost (YLLs) and years lost due to disability (YLDs), representing the overall disease burden of premature death and disability due to a certain disease. Thus, with a stable prevalence, higher DALYs represent the worse quality of care.

Prevalence-to-Incidence ratio: It was calculated by dividing prevalence by incidence. When the incidence is similar, the higher the prevalence means the better ability of averting death.

YLL-to-YLD ratio: It was calculated by dividing YLL by YLD. Higher YLL and lower YLD denote worse situation in health quality, where patients cease earlier than live longer. It reflects the ability of the health system to postpone patients' deaths after diagnoses.

Calculation of QCI

Using data among documented locations over the period of 1990 to 2019, the four ratios above were combined by conducting the principal component analysis (PCA). The first component of PCA was extracted for each combination of age- and sex-group in a reiterate way (age groups includes: 20-24 years, 25-29 years, 30-35 years, 36-39 years, 40-45 years, 46-49 years, 50-54 years, 55-59 years 60-64 years, 65-69 years, 70-74 years, 75-79 years, 80+ years, and age-standardized; sex groups include male, female and both).

In the current research, we conducted post-hoc analyses to justify the rationale to use the first component of PCA to composite the four ratios to represent one characteristic. Taking age-standardized both sex population as an example, results showed that the slop of MIR, DALYs to prevalence, prevalence to incidence and YLL to YLD with the first component reached to 0.99, 0.98, 0.99 and -0.99, respectively. As for the contribution of the four variables (MIR, DALYs to prevalence, prevalence to incidence and YLL to YLD) to the first component of PCA, it was 25.07, 24.85, 24.91 and 25.16 respectively. The eigenvalue of the first component was 3.93. In addition, the first component of PCA could explain a range from 96.93% to 99.28% variations among all sex- and age combination groups of data. Combined all these, it showed an optimal ability to explain the variability in multiple myeloma with the first PCA composite (QCI).

The biplots of the age-standardized group for both sexes, female, and male for all analyzed countries and territories across the 1990-2019, which visually suggested that the data are well-approximated by the first principal component.

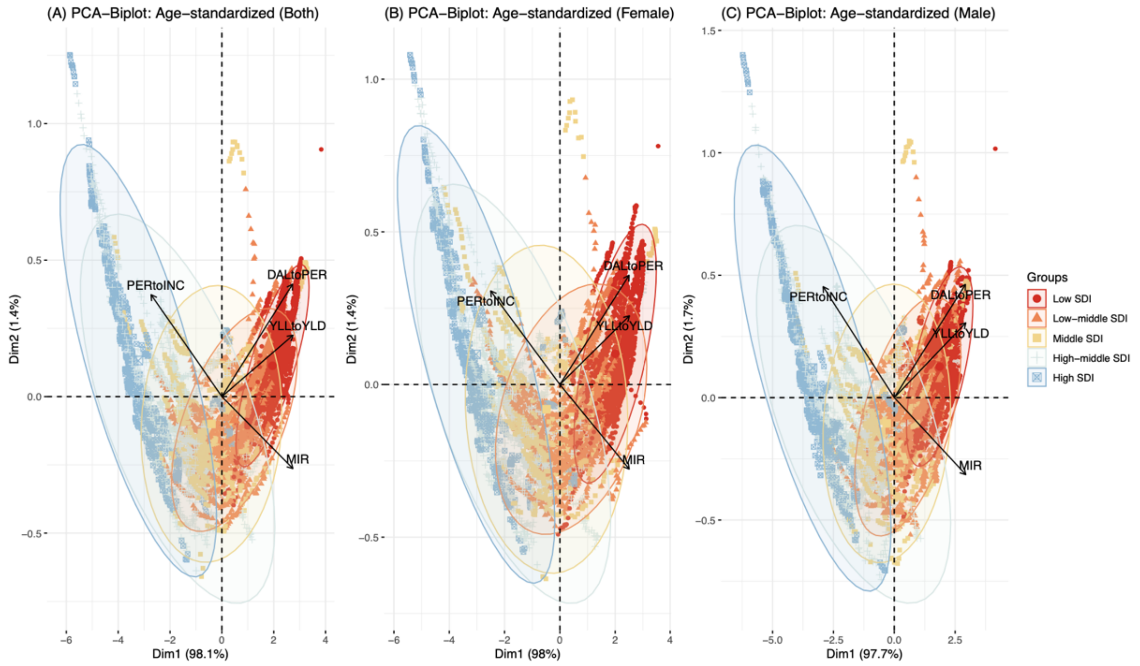

Supplementary Methods Figure.1 Biplot resulting from PCA for age-standardized (A) both sexes, (B) female, (C) male groups; point colours represent socio-demographic status grouped by socio-demographic index (SDI). Explained variance for the first and the second principal components (PC) are displayed. X-axis is PC1

(Dim.1), and y-axis is PC2 (Dim.2). PERtoINC: prevalence to incidence ratio; MIR: mortality to incidence ratio; DALYto PER: DALY to Prevalence Ratio; YLLtoYLD: YLL to YLD ratio.

To test the reliability of using the first PC, we furthelry perfomed a bootstrapping analysis (500 replicates) to see the uncertainty of the explanatory power of the PC1 (PC2 also display in the Table for reference).[4] Simulated variables in the 500 replicated datasets were randomly selected from the actual data, which are normally distributed with the mean and standard deviation determined by the actual data. By repeating the PCA using the 500 bootstrapped datasets, we computed the mean explained variance of the PC1, and its standard error. The analysis was conducted by using 'modelr' and 'FactoMineR' packages in R software. The results are displayed in the **Supplementary methods Table 1** below.

The bootstrapped results showed that, PC1 explained 98.27±0.032% variance in the age-standardized dataset for both sexes, 98.18±0.039% for female, and 97.90±0.038% for male. This indicated the high reliability and explanatory power of PC1.

Supplementary methods Table 1. The actual explained variance and the standard deviation of the he first two principal components estimated with the bootstrap procedure (n=500 bootstrap iterations).

Age-standardized	Principle components (PC)	Variance (%)	Standard deviation of variance
Both sexes	PC1	98.27	0.032
	PC2	1.30	0.025
Female	PC1	98.18	0.039
	PC2	1.28	0.025
Male	PC1	97.90	0.038
	PC2	1.58	0.031

To ease the interpretation and comparison, the loading scores of the first component of PCA were calculated, and rescaled to 0 to 100 spectra following the formula showing below.

$$QCI\ (x) = \frac{PCA_{score}\ (x) - min\ \ PCA_{score}}{max\ PCA_{score}\ - min\ \ PCA_{score}} \times 100$$

In this formular, x means the specific data point of certain age (including age-specific and age-standardized data), sex, location and year; $min\ PCA_{score}$, and $max\ PCA_{score}$ denote the minimum PCA score and the maximum PCA score in the same age, sex, location and year with x.

The higher the QCI is, the better the quality of care is.

2. Assessment of gender disparity ratio (GDR)[1]

$$GDR(x) = \frac{QCI\ female\ (x)}{QCI\ male\ (x)}$$

In this formular, x means the specific data point of certain age, location and year. GDR equals to 1 denotes the absolute equity in QCI between female and male.

REFERENCES:

1 Mohammadi E, Ghasemi E, Azadnajafabad S, Rezaei N, Saeedi Moghaddam S, Ebrahimi Meimand S, et al. A global, regional, and national survey on burden and Quality of Care Index (QCI) of brain and other central nervous system cancers; global burden of disease systematic analysis 1990-2017. PLoS One. 2021;16:e0247120.

2 Keykhaei M, Masinaei M, Mohammadi E, Azadnajafabad S, Rezaei N, Saeedi Moghaddam S, et al. A global, regional, and national survey on burden and Quality of Care Index (QCI) of hematologic malignancies; global burden of disease systematic analysis 1990-2017. Exp Hematol Oncol. 2021;10:11.

3 Aryannejad A, Tabary M, Ebrahimi N, Mohammadi E, Fattahi N, Roshani S, et al. Global, regional, and national survey on the burden and quality of care of pancreatic cancer: a systematic analysis for the Global Burden of Disease study 1990–2017. Pancreatology. 2021;21:1443-50.

4 Gomarasca U, Migliavacca M, Kattge J, Nelson JA, Niinemets Ü, Wirth C, et al. Leaf-level coordination principles propagate to the ecosystem scale. Nature communications. 2023;14:3948.

Table S1. Estimated number of multiple myeloma incidence and deaths from 2020 to 2030

Year	Projected incidence cases			Projected deaths cases		
	Both	Female	Male	Both	Female	Male
2020	160276.43 (154613.63, 165939.87)	73183.44 (70198.44, 76169.05)	87317.79 (83913.43, 90722.82)	116910.56 (112745.67, 121076.07)	54570.73 (52233.47, 56908.58)	62479.6 (59864.47, 6 5095.38)
2021	165106.5 (158283.92, 1 71929.71)	75317.12 (71793.7, 7 8841.16)	90026.24 (85997.96, 94055.19)	120479.08 (115495.65, 125463.14)	56175.01 (53430.83, 58919.79)	64443.45 (61390.56, 67496.98)
2022	170008.1 (161428.9, 17 8587.93)	77484.62 (73149.87, 81819.99)	92753.67 (87784.03, 97724)	124085.03 (117868.37, 130302.32)	57801.86 (54448.25, 61156.08)	66411.1 (62702.07, 7 0120.77)
2023	175110.22 (164201.49, 186019.6)	79737.9 (74325.01, 8 5151.41)	95584.93 (89361.49, 101809.08)	127839.45 (119988.26, 135691.28)	59495.82 (55333.74, 63658.52)	68455.64 (63871.55, 73040.39)
2024	180471.82 (166684.51, 194259.79)	82104.85 (75356.36, 88853.98)	98571.78 (90789.88, 106354.39)	131822.77 (121948.27, 141697.9)	61290.53 (56126.13, 66455.54)	70633.91 (64958.09, 76310.4)
2025	185981.94 (168786.63, 203177.9)	84535.99 (76204.89, 92867.73)	101657.39 (92021.63 , 111293.86)	135949.17 (123675.34, 148223.63)	63149.29 (56795.61, 69503.58)	72897.8 (65919.92, 7 9876.34)
2026	191564.25 (170471.26, 212657.91)	87000.36 (76857.28, 97144.09)	104786.95 (93024.35 , 116550.27)	140146.06 (125123, 15 5169.76)	65044.63 (57326.44, 72763.44)	75199.3 (66723.6, 83 675.67)
2027	197196.99 (171732.02, 222662.63)	89483.44 (77306.67, 101660.84)	107936.01 (93783.42 , 122089.33)	144370.27 (126260.34, 162480.86)	66958.26 (57705.87, 76211.27)	77506.38 (67345, 876 68.44)
2028	203013.05 (172678.72, 233348.06)	92041.76 (77599.76, 106484.41)	111187.08 (94367.45 , 128007.46)	148742.23 (127188.4, 170296.72)	68942.29 (57976.64, 79908.56)	79893.38 (67846.9, 9 1940.55)
2029	209087.18 (173347.29, 244827.75)	94708.86 (77753.5, 1 11664.87)	114594.09 (94806.73 , 134382.21)	153348.15 (127960.69, 178736.27)	71031.57 (58158.64, 83905.12)	82416.25 (68265.96, 96567.24)
2030	215313.37 (173614.57, 257012.86)	97431.4 (77710.14, 1 17153.31)	118105.3 (95038.55, 141172.8)	158095.13 (128473.49, 187717.43)	73179.92 (58202.25, 88158.23)	85025.14 (68546.63, 101504.36)

Table S2. Frontier age standardized quality of care index (QCI), and effective difference by countries and territories.

Location	HAQ	Sociodemographic index (SDI)	SDI quintile b	Age standardized QCI	Frontier QCI	Effective difference	Effective difference rank (Age standardized QCI rank)
Afghanistan	28.9	0.343	Low	19.66	31.63	11.97	35 (181)
Albania	67.5	0.681	Middle	46.83	77.59	30.76	143 (90)
Algeria	58.7	0.652	Middle	45.25	74.25	29	136 (97)
American Samoa	45.5	0.712	High-middle	35.37	80.09	44.72	181 (117)
Andorra	89.1	0.894	High	77.11	100	22.89	97 (18)
Angola	29.3	0.47	Low-middle	21.03	44.13	23.1	100 (174)
Antigua and Barbuda	58.2	0.743	High-middle	60.49	83.78	23.29	103 (45)
Argentina	59.9	0.708	High-middle	52.01	79.8	27.79	129 (69)
Armenia	63.2	0.689	Middle	44.62	78.38	33.76	149 (100)
Australia	90.2	0.839	High	89.79	100	10.21	26 (5)
Austria	88	0.849	High	75.15	100	24.85	114 (22)
Azerbaijan	53.3	0.683	Middle	32.84	77.81	44.97	184 (125)
Bahamas	52.6	0.796	High-middle	55.86	94.01	38.15	165 (53)
Bahrain	67.6	0.751	High-middle	51.08	85.13	34.05	152 (74)
Bangladesh	44.1	0.483	Low-middle	32.21	45.79	13.58	43 (127)
Barbados	59	0.742	High-middle	64.08	83.59	19.51	73 (40)

Belarus	71.2	0.745	High-middle	73.17	84.13	10.96	31 (28)
Belgium	86.6	0.851	High	73.87	100	26.13	118 (25)
Belize	49.5	0.603	Low-middle	52.14	63.25	11.11	33 (68)
Benin	31.4	0.352	Low	22.52	32.51	9.99	21 (165)
Bermuda	77.4	0.813	High	80.7	96.83	16.13	59 (12)
Bhutan	42.1	0.455	Low-middle	33.33	42.13	8.8	17 (121)
Bolivia (Plurinational State of)	40.5	0.566	Low-middle	33.09	54.17	21.08	85 (123)
Bosnia and Herzegovina	68.6	0.718	High-middle	42.74	80.57	37.83	162 (105)
Botswana	37.5	0.634	Middle	33.64	70.63	36.99	160 (120)
Brazil	53	0.64	Middle	48.2	71.89	23.69	106 (84)
Brunei Darussalam	57	0.823	High	47.95	98.45	50.5	189 (85)
Bulgaria	64.9	0.764	High-middle	64	87.67	23.67	105 (41)
Burkina Faso	28.5	0.257	Low	22.17	23.26	1.09	4 (166)
Burundi	25.8	0.284	Low	17.25	25.63	8.38	15 (191)
Cabo Verde	50.2	0.525	Low-middle	39.82	48.7	8.88	18 (109)
Cambodia	38	0.469	Low-middle	29.93	44	14.07	47 (132)
Cameroon	33.7	0.49	Low-middle	24.62	46.47	21.85	89 (152)
Canada	90.7	0.873	High	88.14	100	11.86	34 (6)
Central African Republic	15.2	0.274	Low	10.74	24.69	13.95	45 (195)
Chad	23.8	0.238	Low	18.2	22.12	3.92	8 (185)

Chile	70.9	0.759	High-middle	60.07	86.69	26.62	123 (46)
China	70.2	0.686	Middle	62.12	78.11	15.99	58 (44)
Colombia	61.1	0.633	Middle	56.87	70.41	13.54	42 (51)
Comoros	31.8	0.455	Low-middle	21.38	42.13	20.75	81 (172)
Congo	34	0.568	Low-middle	21.79	54.6	32.81	147 (169)
Costa Rica	64.7	0.68	Middle	57.36	77.48	20.12	77 (49)
Côte d'Ivoire	34.3	0.408	Low	21.91	36.93	15.02	50 (168)
Croatia	81.4	0.794	High-middle	66.68	93.67	26.99	125 (37)
Cuba	66.2	0.668	Middle	71.61	76.41	4.8	12 (32)
Cyprus	86.2	0.841	High	73.83	100	26.17	120 (27)
Czechia	81.5	0.828	High	57.06	99.27	42.21	179 (50)
Democratic People's Republic of Korea	50.1	0.558	Low-middle	39.08	52.59	13.51	41 (111)
Democratic Republic of the Congo	29	0.382	Low	19.07	34.87	15.8	56 (183)
Denmark	85.5	0.89	High	81.57	100	18.43	66 (10)
Djibouti	32.6	0.459	Low-middle	23.16	42.67	19.51	72 (158)
Dominica	45.2	0.729	High-middle	47.71	81.67	33.96	151 (86)
Dominican Republic	45.4	0.592	Low-middle	50.5	60.44	9.94	20 (77)
Ecuador	52.9	0.64	Middle	45.48	71.89	26.41	122 (96)
Egypt	51.6	0.658	Middle	37.18	75.36	38.18	166 (115)

El Salvador	54.7	0.573	Low-middle	50.76	55.68	4.92	13 (76)
Equatorial Guinea	42.4	0.685	Middle	27.4	78.01	50.61	190 (144)
Eritrea	25.6	0.396	Low	17.71	35.91	18.2	64 (189)
Estonia	76.4	0.835	High	77.67	100	22.33	94 (16)
Eswatini	32.5	0.577	Low-middle	22.65	56.59	33.94	150 (164)
Ethiopia	31.2	0.343	Low	21.47	31.63	10.16	24 (171)
Fiji	38.7	0.664	Middle	28.57	76.08	47.51	187 (138)
Finland	87.7	0.856	High	80.06	100	19.94	76 (14)
France	88	0.834	High	73.85	100	26.15	119 (26)
Gabon	39.6	0.656	Middle	26.82	75.03	48.21	188 (146)
Gambia	34.7	0.399	Low	23.13	36.15	13.02	38 (159)
Georgia	57.7	0.702	High-middle	39.13	79.38	40.25	173 (110)
Germany	87	0.898	High	86.22	100	13.78	44 (7)
Ghana	36.1	0.557	Low-middle	27.21	52.43	25.22	115 (145)
Greece	83.9	0.794	High-middle	72.88	93.67	20.79	82 (29)
Greenland	62.7	0.761	High-middle	53.37	87.08	33.71	148 (64)
Grenada	50.4	0.669	Middle	53.48	76.49	23.01	99 (62)
Guam	56.5	0.813	High	43.22	96.83	53.61	191 (104)
Guatemala	43.6	0.526	Low-middle	38.69	48.77	10.08	23 (112)
Guinea	25.7	0.325	Low	19.43	29.89	10.46	29 (182)

Guinea-Bissau	24.3	0.355	Low	20.2	32.78	12.58	37 (178)
Guyana	37.2	0.618	Middle	46.48	66.97	20.49	79 (92)
Haiti	24.5	0.432	Low	29.18	39.26	10.08	22 (136)
Honduras	40	0.496	Low-middle	37.33	46.93	9.6	19 (114)
Hungary	74.4	0.791	High-middle	48.3	93.16	44.86	182 (82)
Iceland	93.1	0.869	High	80.16	100	19.84	75 (13)
India	39.2	0.566	Low-middle	31.31	54.17	22.86	96 (130)
Indonesia	40.9	0.66	Middle	30.7	75.64	44.94	183 (131)
Iran (Islamic Republic of)	63.7	0.67	Middle	53.44	76.56	23.12	101 (63)
Iraq	57.4	0.671	Middle	45.99	76.64	30.65	142 (95)
Ireland	90.1	0.867	High	77.63	100	22.37	95 (17)
Israel	83.1	0.803	High-middle	70.46	95.21	24.75	113 (34)
Italy	89.6	0.801	High-middle	90.89	94.87	3.98	9 (4)
Jamaica	55.5	0.684	Middle	55.81	77.91	22.1	92 (54)
Japan	87.5	0.87	High	76.79	100	23.21	102 (20)
Jordan	65.1	0.731	High-middle	51.45	81.92	30.47	141 (71)
Kazakhstan	59.5	0.723	High-middle	41.4	81.03	39.63	170 (108)
Kenya	33.4	0.508	Low-middle	18.08	47.66	29.58	137 (187)
Kiribati	24.2	0.527	Low-middle	18.75	48.84	30.09	139 (184)
Kuwait	77	0.851	High	62.85	100	37.15	161 (42)

Kyrgyzstan	54.2	0.596	Low-middle	33.84	61.45	27.61	128 (119)
Lao People's Democratic Republic	33	0.49	Low-middle	24.91	46.47	21.56	87 (151)
Latvia	69.5	0.82	High	69.66	97.96	28.3	132 (35)
Lebanon	68.2	0.708	High-middle	59.56	79.8	20.24	78 (47)
Lesotho	26.3	0.507	Low-middle	17.68	47.6	29.92	138 (190)
Liberia	35.7	0.37	Low	22.89	33.99	11.1	32 (162)
Libya	59.5	0.709	High-middle	44.24	79.87	35.63	156 (102)
Lithuania	67.9	0.843	High	71.4	100	28.6	134 (33)
Luxembourg	87.5	0.895	High	74.37	100	25.63	116 (23)
Madagascar	29	0.396	Low	18.09	35.91	17.82	63 (186)
Malawi	29.9	0.384	Low	20.63	35.01	14.38	48 (177)
Malaysia	55.4	0.737	High-middle	46.38	82.76	36.38	158 (93)
Maldives	60.7	0.562	Low-middle	50.89	53.3	2.41	7 (75)
Mali	29.6	0.263	Low	22.15	23.73	1.58	6 (167)
Malta	85.1	0.801	High-middle	73.98	94.87	20.89	84 (24)
Marshall Islands	32.1	0.544	Low-middle	23.42	50.48	27.06	127 (156)
Mauritania	42.1	0.496	Low-middle	28.27	46.93	18.66	68 (141)
Mauritius	56.7	0.705	High-middle	47.6	79.59	31.99	146 (88)
Mexico	52.5	0.649	Middle	51.78	73.67	21.89	90 (70)

Micronesia (Federated States of)	35.6	0.58	Low-middle	28.5	57.34	28.84	135 (140)
Mongolia	47.4	0.606	Low-middle	28.51	64.02	35.51	155 (139)
Montenegro	76.1	0.791	High-middle	47.63	93.16	45.53	186 (87)
Morocco	48.5	0.548	Low-middle	37.72	51.02	13.3	40 (113)
Mozambique	25.1	0.307	Low	17.75	27.98	10.23	27 (188)
Myanmar	37.5	0.521	Low-middle	28.78	48.43	19.65	74 (137)
Namibia	39.9	0.612	Middle	29.62	65.52	35.9	157 (135)
Nepal	38.8	0.422	Low	27.77	38.29	10.52	30 (143)
Netherlands	91.1	0.883	High	77.03	100	22.97	98 (19)
New Zealand	85.5	0.84	High	99.29	100	0.71	3 (1)
Nicaragua	52.2	0.517	Low-middle	49.9	48.18	-1.72	1 (79)
Niger	26.5	0.162	Low	20.19	19.67	-0.52	2 (179)
Nigeria	31.6	0.515	Low-middle	25.94	48.06	22.12	93 (149)
North Macedonia	67.7	0.744	High-middle	44.26	83.96	39.7	171 (101)
Northern Mariana Islands	55.7	0.771	High-middle	47.09	89.1	42.01	177 (89)
Norway	90.4	0.913	High	81	100	19	70 (11)
Oman	67.5	0.783	High-middle	53.22	91.62	38.4	167 (65)
Pakistan	32.4	0.449	Low	24.48	41.31	16.83	61 (153)
Palestine	57.3	0.588	Low-middle	44.96	59.43	14.47	49 (98)
Panama	59.3	0.686	Middle	53.6	78.11	24.51	110 (61)

Papua New Guinea	31.4	0.394	Low	20.06	35.76	15.7	54 (180)
Paraguay	51.7	0.638	Middle	46.81	71.48	24.67	111 (91)
Peru	60	0.648	Middle	49.86	73.47	23.61	104 (80)
Philippines	40.8	0.623	Middle	33.18	68.14	34.96	154 (122)
Poland	73.2	0.802	High-middle	25.31	95.04	69.73	194 (150)
Portugal	83.9	0.743	High-middle	27.8	83.78	55.98	192 (142)
Puerto Rico	70.6	0.814	High	76.28	96.99	20.71	80 (21)
Qatar	73.7	0.83	High	55.55	99.59	44.04	180 (56)
Republic of Korea	86.3	0.878	High	72.06	100	27.94	130 (30)
Republic of Moldova	63.7	0.696	High-middle	62.71	78.95	16.24	60 (43)
Romania	69.7	0.76	High-middle	46.31	86.89	40.58	175 (94)
Russian Federation	67.6	0.805	High-middle	54.19	95.55	41.36	176 (60)
Rwanda	31.8	0.429	Low	23.03	38.9	15.87	57 (161)
Saint Lucia	52.8	0.67	Middle	55.72	76.56	20.84	83 (55)
Saint Vincent and the Grenadines	47.9	0.627	Middle	49.61	69.06	19.45	71 (81)
Samoa	43.7	0.641	Middle	35.16	72.09	36.93	159 (118)
Sao Tome and Principe	41.4	0.502	Low-middle	29.73	47.32	17.59	62 (133)
Saudi Arabia	63.3	0.805	High-middle	55	95.55	40.55	174 (58)
Senegal	34	0.389	Low	23.38	35.38	12	36 (157)

Serbia	72.2	0.767	High-middle	10.98	88.28	77.3	195 (194)
Seychelles	52.8	0.724	High-middle	41.65	81.13	39.48	169 (107)
Sierra Leone	30.9	0.347	Low	21.72	32.03	10.31	28 (170)
Singapore	86.2	0.861	High	71.85	100	28.15	131 (31)
Slovakia	73.4	0.812	High	92.52	96.67	4.15	10 (2)
Slovenia	87.8	0.84	High	57.92	100	42.08	178 (48)
Solomon Islands	30.3	0.407	Low	26.67	36.84	10.17	25 (148)
Somalia	16.7	0.081	Low	12.11	13.43	1.32	5 (193)
South Africa	44.6	0.678	Middle	31.96	77.25	45.29	185 (128)
South Sudan	29.1	0.363	Low	15.22	33.45	18.23	65 (192)
Spain	89.7	0.767	High-middle	84.01	88.28	4.27	11 (9)
Sri Lanka	60.5	0.69	High-middle	50.08	78.47	28.39	133 (78)
Sudan	43.9	0.515	Low-middle	32.85	48.06	15.21	52 (124)
Suriname	43	0.636	Middle	44.06	71.06	27	126 (103)
Sweden	90.4	0.872	High	68.38	100	31.62	145 (36)
Switzerland	92.6	0.929	High	84.87	100	15.13	51 (8)
Syrian Arab Republic	60.2	0.619	Middle	48.24	67.21	18.97	69 (83)
Taiwan (Province of China)	78	0.868	High	65.89	100	34.11	153 (38)
Tajikistan	42.5	0.539	Low-middle	23.57	49.9	26.33	121 (155)
Thailand	62.5	0.687	Middle	51.34	78.2	26.86	124 (72)

Timor-Leste	35.7	0.514	Low-middle	26.8	48	21.2	86 (147)
Togo	33.5	0.417	Low	23.85	37.82	13.97	46 (154)
Tonga	45.8	0.636	Middle	32.49	71.06	38.57	168 (126)
Trinidad and Tobago	52.9	0.757	High-middle	55.32	86.3	30.98	144 (57)
Tunisia	63.9	0.672	Middle	52.88	76.71	23.83	107 (67)
Turkey	64.8	0.748	High-middle	54.43	84.63	30.2	140 (59)
Turkmenistan	48.7	0.67	Middle	36.66	76.56	39.9	172 (116)
Uganda	32.4	0.404	Low	20.8	36.57	15.77	55 (176)
Ukraine	63.1	0.736	High-middle	64.12	82.6	18.48	67 (39)
United Arab Emirates	58.8	0.88	High	42.33	100	57.67	193 (106)
United Kingdom	83.3	0.847	High	91.39	100	8.61	16 (3)
United Republic of Tanzania	32.5	0.423	Low	22.88	38.36	15.48	53 (163)
United States of America	80.6	0.859	High	77.91	100	22.09	91 (15)
United States Virgin Islands	56.7	0.799	High-middle	56.64	94.52	37.88	163 (52)
Uruguay	64.7	0.697	High-middle	53.01	79.02	26.01	117 (66)
Uzbekistan	49	0.631	Middle	31.92	69.97	38.05	164 (129)
Vanuatu	31.1	0.485	Low-middle	21.28	46	24.72	112 (173)
Venezuela (Bolivarian Republic of)	54.1	0.607	Low-middle	51.19	64.28	13.09	39 (73)
Viet Nam	55.6	0.617	Middle	44.9	66.73	21.83	88 (99)

Yemen	39.3	0.412	Low	29.69	37.31	7.62	14 (134)
Zambia	31.6	0.505	Low-middle	23.1	47.49	24.39	109 (160)
Zimbabwe	28.6	0.476	Low-middle	20.9	44.93	24.03	108 (175)

HAQ–Health-care access and quality index.
*Pearson correlation coefficient between HAQ and QCI in 2019 is 0.90, $P<0.001$.
†SDI quintile is classified as low SDI [0, 0.455], low-middle SDI (0.455, 0.608], middle SDI (0.608, 0.690], high-middle SDI (0.690, 0.805], and high SDI (0.805, 1].

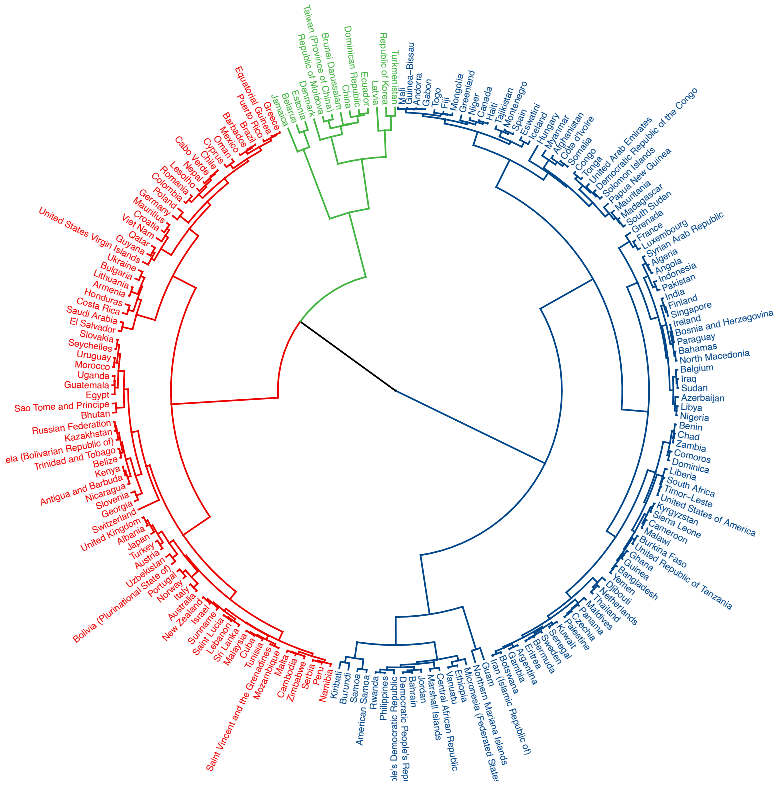

Figure S1. The hierarchical clustering analysis of estimated annual percentage changes (EAPC) of age-standardized prevalence rate for multiple myeloma among 195 countries and territories.

The blue cluster represents remained stable or low increase (104 geographies); the red cluster represents middle increase (78 geographies); the green cluster represents high increase (13 geographies).

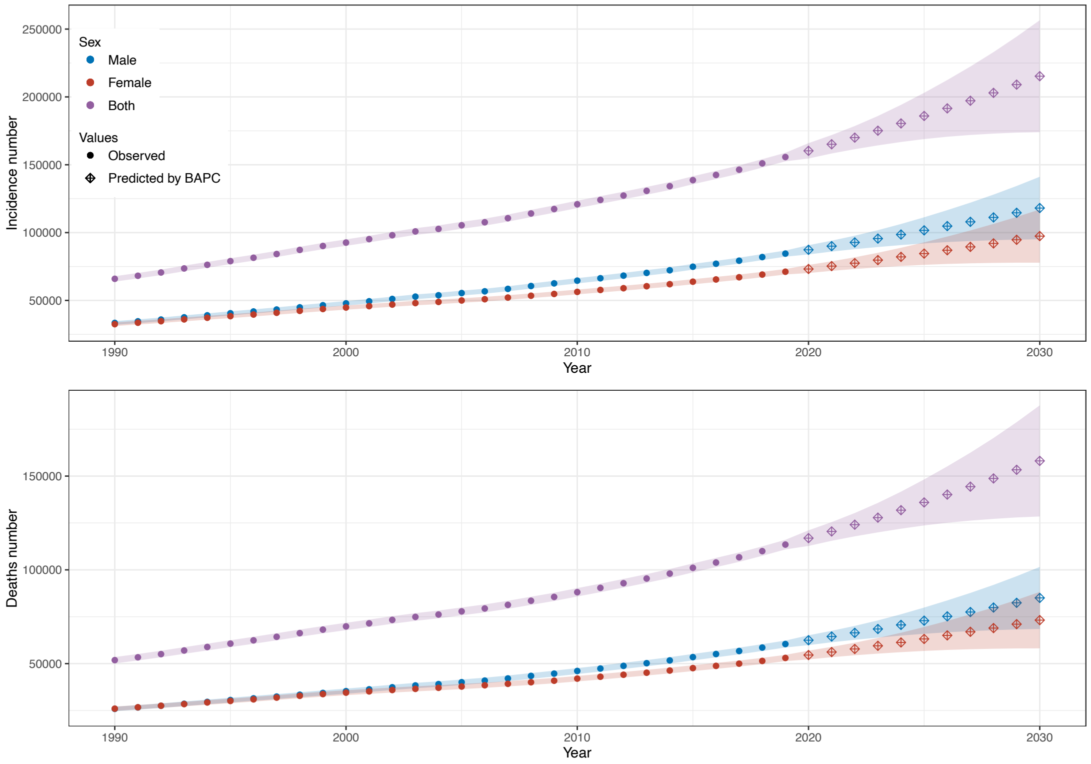

Figure S2. Trends in multiple myeloma sex-specific incidence and deaths of global from 1990-2030.

Dots represent observed number of incident cases and deaths; Square crosses represent predicted number of incident cases and deaths by Bayesian age-period-cohort models (BAPC); For reference, shading represents 95% confidence interval of the predicted results.

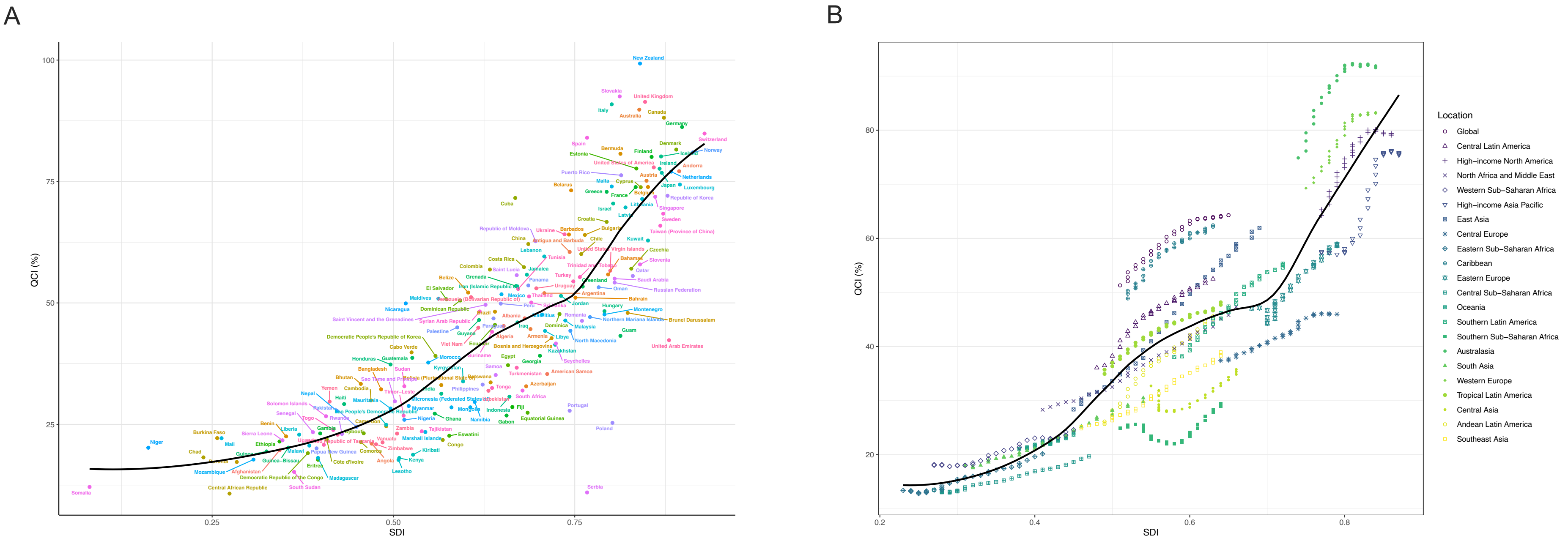

Figure S3. The association between quality of care index (QCI) and socio-demographic index (SDI). **Panel A**. Countries. **Panel B**. Regions.

The smoothed line in black depicted by using loess regression shows the possible relationship between SDI and QCI; The Pearson correlation coefficient between SDI and QCI on national level was 0.829.

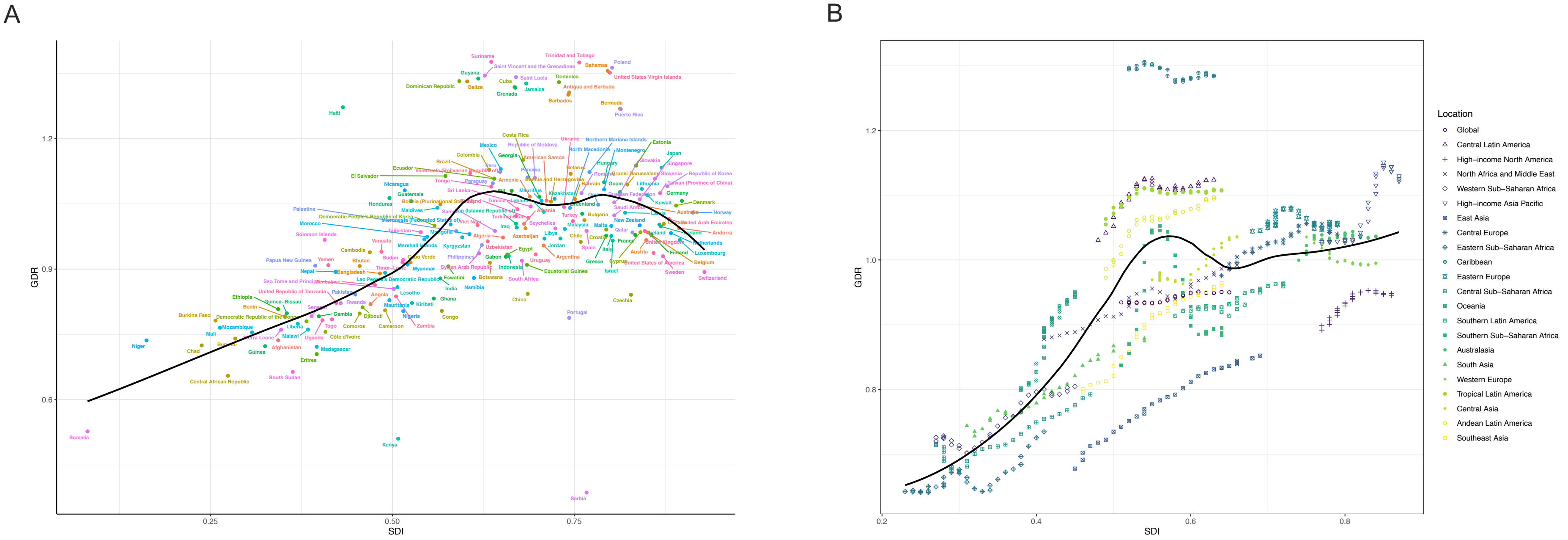

Figure S4. The association between gender disparity ratio (GDR) and socio-demographic index (SDI). **Panel A**. Countries. **Panel B**. Regions.

The smoothed line in black depicted by using loess regression shows the possible relationship between SDI and GDR; The Pearson correlation coefficient between SDI and GDR on national level was 0.554.

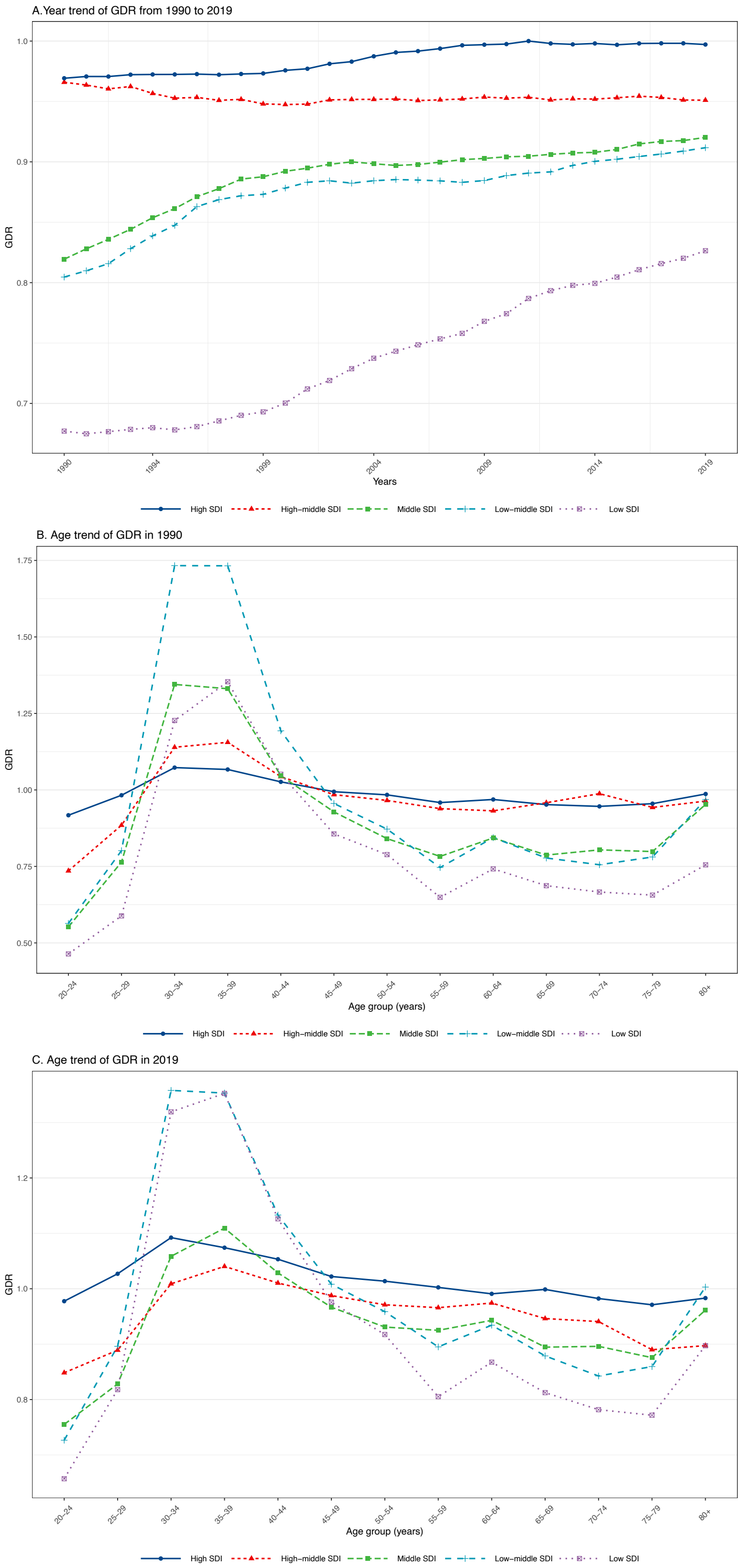

Figure S5. Gender disparity ratio (GDR) by socio-demographic index (SDI) quintiles. **Panel A.** Temporal GDR from 1990 to 2019. **Panel B.** Age trend of GDR in 1990. **Panel C.** Age trend of GDR in 2019.
